# DEMENTIA IMMERSION SIMULATION EXPERIENCE (DISE): ELICITING EMPATHY, IMPROVING COMMUNICATION SKILLS IN DEMENTIA

**DOI:** 10.1093/geroni/igz038.3226

**Published:** 2019-11-08

**Authors:** Gabriela Zaragoza, Jaime Cruz-Martinez, Paula Reilly, Jeanette Ross, Michael J Mader, Lyda C Arevalo-Flechas

**Affiliations:** 1 The University of Texas Health Science Center San Antonio, San Antonio, Texas, United States; 2 The Long School of Medicine at UT Health San Antonio, San Antonio, Texas, United States; 3 Health Careers High School, San Antonio, Texas, United States; 4 University of Texas Health Science Center San Antonio, San Antonio, Texas, United States; 5 South Texas Veterans Health Care System, Dept of Research, San Antonio, Texas, United States; 6 South Texas Veterans Heath Care System - Geriatrics and Extended Care Service, San Antonio, United States

## Abstract

Dementia awareness training alone does not improve care or outcomes for patients living with dementia. Effective dementia education programs for family caregivers and healthcare providers can lead to improved care practices and patient outcomes. The Dementia Immersion Simulation Experience (DISE) is a face-to-face 2-hour educational program that includes simulation, videos, a virtual reality station, group debriefing, and a didactic session delivered by faculty with dementia caregiving expertise. The purpose of this project was to evaluate the effectiveness of DISE in a group of 48 interdisciplinary healthcare providers, trainees and administrative staff. A program evaluation and pre and post knowledge questionnaires were administered. Prior to the activity, the mean score of all participants was 8.85. After the activity, the mean score was 10.1 (p<0.0001). 35.4% of all participants were well informed on dementia before DISE and 70.8% were well informed after the activity (p <0.0005). Qualitative analysis of the comments section of the program evaluation showed that 95% of the participants mentioned empathy for those living with dementia. Participants rated DISE on a scale of 1 (Strongly Disagree) to 5 (Strongly Agree) across ten categories, covering objectives, relevance, effectiveness, and value of the learning experience. Over 95% of respondents agreed or better (score = 4 or 5) with each evaluation statement and at least 85% strongly agreed with each statement. The evaluation scores are further evidence of an effective program. DISE is an effective tool to teach and support family caregivers, healthcare workers, and healthcare professionals and trainees.

